# The genetic characterization of germplasm and identification of the litter size trait associated candidate genes in Dexin mutton and fine-wool sheep

**DOI:** 10.3389/fgene.2024.1457634

**Published:** 2024-08-14

**Authors:** Mengting Zhu, Pengfei Li, Weiwei Wu, Wenxin Zheng, Juncheng Huang, Hanikzi Tulafu, Changchun Lin, Weikun Tao, Qi Aladaer

**Affiliations:** ^1^ College of Animal Science, Xinjiang Agricultural University, Urumqi, China; ^2^ Institute of Animal Science, Xinjiang Academy of Animal Sciences, Urumqi, China

**Keywords:** Dexin mutton and fine-wool sheep, litter size trait, selection signature, candidate genes, whole-genome resequencing

## Abstract

Xinjiang is a major province of sheep breeding in China, which plays an important role in meeting people’s needs for meat products, increasing farmers’ income and sustainable development of animal husbandry. However, the genetic differentiation relationship between breeds was not clear, and most sheep had low fecundity, which seriously restricted the efficient development of sheep industry. Therefore, this study used the whole genome resequencing to detect the genetic variation of Dexin mutton and fine-wool sheep, explored the selected regions and important genes of the litter size traits, analyzed the genetic mechanism of reproductive traits, and provided new insights for the high fecundity breeding of sheep. A total of 5,236.338 G genome data and 35,884,037 SNPs were obtained. Furthermore, we identified 39 selection signals spanning candidate genes, 99 genes were significantly associated related to growth, reproduction and immunity, among which, *BRIP1, BMPR1B, BMP4, NGF*, etc. genes, and MAKP signaling pathway, Fanconi anemia pathway and Thyroid hormone signaling pathway and other signaling pathways were significantly correlated with litter size trait. Among them, we identified *NGF, TrKA* and *BRIP1* genes was the important genes for sheep litter size traits and the mutation frequencies of 9 SNPs in *BRIP1* gene were significantly different in domestic sheep in the world. The research provided new insights for the breeding of self-cultivated meat fine-wool sheep.

## Background

Sheep have domesticated about 11,000 years ago in the Fertile Crescent and provided necessary basis for human economy and culture (Alberto et al., 2018). China is a large agricultural country, animal husbandry was one of the main industries and played an important role in national economic and social development. Mutton is favored by consumers because of its unique flavor, high protein and low cholesterol. In recent years, the shortage of mutton-based sheep products had led to a straight upward trend mutton prices in China. The contradiction between the rapid rised in Chinese mutton demand and the low production capacity of domestic mutton had become increasingly prominent. Improving the production performance of ewes and mutton production had become a major industrial demand at this stage. Reproductive traits were the most important production traits in important economic traits (reproduction, meat and fur) of sheep (Wang et al., 2019; Zhang et al., 2022; Zhong et al., 2024). Whereas, Multiple lambing (high fecundity) was an important basis for efficient breeding and sustainable development of sheep production industry. With the upgrading of sequencing technology, the cost of sequencing had also been reduced. In the study of major economic animals, the whole genome resequencing analysis strategy had been widely used. More and more researchers had carried out whole genome sequencing research in order to identify a large number of candidate genes related to important economic traits of candidate groups at the genomic level. Prof. Li’s team deep resequencing of 248 sheep including the wild ancestor (*O. orientalis*), landraces, and improved breeds individuals from all over the world at an average sequencing depth of ∼25.7 ×, providing insights into the demographic history of sheep and a valuable genomic resource for future genetic studies and improved genome-assisted breeding of sheep and other domestic animals (Li et al., 2020). Liu and his team revealed the genetic basis of year-round estrus, drought tolerance, hypoxia resistance, and cold tolerance traits of Xinjiang sheep breeds resistance, and cold tolerance traits of Xinjiang sheep breeds (Zhang et al., 2023). Furthermore, Li et al. (2022) revealed that *PAPSS2* was used to high-altitude adaptability in Tibetan goats, following interspecific introgression and natural selection. Similarly, Zhu et al. (2023) provides insights into the microevolution and identifies genes associated with reproduction traits via whole genome resequencing.

A fine breed was an important foundation to ensure the healthy development of sheep industry and the key to enhance the core competitiveness of sheep industry. The project team members had gone through 16 years (2008–2023) in the early stage, with German Merino sheep as the male parent and Chinese Merino sheep as the female parent. Through three stages of progressive hybridization, cross fixation and breeding improvement, after four generations of continuous breeding, a new breed of fine-wool sheep with uniform body shape, stable genetic performance and suitable for both meat and wool in the northern pastoral area and the farming-pastoral ecotone was bred. The main characteristics are fast growth and development, good meat performance, strong adaptability, resistance to roughage, suitable for feeding in the majority of agricultural and pastoral areas, and high breeding efficiency. At present, the population size of Dexin mutton and fine-wool sheep has reached 84,000, of which the number of core breeding groups and breeding groups has reached 15,000, and the number of families has reached more than 8. In terms of reproductive traits, Dexin mutton and fine-wool sheep reached sexual maturity at 9 months of age, and ewes reached sexual maturity at 8 months of age. The age of ewes was 15 months, and the age of rams was 18 months. The estrous cycle of ewes was 17–21 days, the duration of estrus was 36–48 h, and the gestation period was about 150 days. The lambing rate of multiparous ewes was 130%, and the survival rate of lambs was more than 95%.

Based on this, this study used whole genome resequencing technology to study the selected regions and candidate genes of the litter size trait of Dexin mutton and fine-wool sheep, and identified the specific selection signals. These genes contribute to the reproductive characteristics of the species and deepen our understanding of the unique genomic structure of the species, so as to further improve and sustainable use.

## Materials and methods

### Ethics approval and consent to participate

All animal treatments were according to the recommendation of the Regula-tions for the Administration of Affairs Concerning Experimental Animals of China, and approved by the Animal Care Committee of Xinjiang Agricultura University responsible for overseeing the ethical use of animals in research within the university. All methods are reported in accordance with ARRIVE guidelines for the reporting of animal experiments (2024004).

### Sample collection

Whole-blood samples (5 mL) were collected from 51 Dexin mutton and fine-wool sheep (single-lamb group: 31 and double-lamb group: 20), randomly selected from Keping County, Aksu, southern Xinjiang. DNA was extracted from blood using the TIANamp Blood DNA Kit (TIANGEN, China) according to the manufacturer’s instructions. To investigate the genetic basis of Dexin mutton and fine-wool sheep. The whole-genome sequencing (WGS) 10 × was performed using the Illumina HiSeq2500 Sequencer (Illumina Inc.) by Tianjin Compass Biotechnology Co., Ltd. (Tianjin, China). To obtain high-quality data, reads containing adapters, reads with ≥10% unidentified nucleotides (N) were removed, and low-quality reads (>50% of the read bases with a Phred quality score (i.e., Q-score) ≤5) were also removed.

Additionally, one hundred genomic DNA samples were obtained from healthy Dexin mutton and fine-wool sheep by intravenous blood collection in Tianxin Breeding Farm, Aksu, southern Xinjiang, China (4 years old and weighed 45 ± 3.44 kg). Another one hundred and sixteen genomic DNA samples were obtained from healthy Dexin mutton and fine-wool sheep by intravenous blood collection in Wenquan Breeding Farm, Aksu, southern Xinjiang, China (4 years old and weighed 48 ± 2.94 kg). These sample DNA were used for SNPs validation and application.

### Mapping and variant calling

To obtain high-quality data, the effective sequencing data was compared to the sheep reference genome sequence (ARS-UI Ramb v2.0) by using BWA (V0.7.12) with default parameters (mem -t 4 -k 32 -M) (Li and Durbin, 2009). SAMtools version 1.9 was employed to convert SAM files to the BAM format and subsequently sort the resulting BAM files by contigs (Li et al., 2009). The SNPs were detected by SAMtools (v1.2) with the following parameters “mpileup -m 2 -F 0.002 -d 1,000” (Li et al., 2009). Moreover, to exclude SNP callings errors, variant sites with QD < 2.0, MQ < 20, FS > 60.0 were discarded, then variants were annotated by using ANNOVAR v.21-Jun-2013. The VCF file was obtained by Haplotyper and GVCFtyper, and the data were filtered by PLINK software (v.1.9). After variant calling and obtaining the variant call set, we performed genomic diversity analysis to explore the patterns of genetic variation within populations. The loci with MAF less than 0.05, the loci with markers less than 1e-5 deviating from Hardy-Weinberg equilibrium (--maf 0.05 -hwe 1e-6), and the loci with deletion rate more than 10% were excluded (--geno 0.10). Principal component analysis (PCA) of the 51 samples was conducted by EIGENSOFT (Price et al., 2006). Based on neighbor-joining (NJ) method, phylogenetic tree was constructed by PHYLIP (v.3.6) (Felsenstein, 1989), and displayed by Newick Utilities.

### Genome-wide selective sweep regions

In this study, Nucleotide diversity (π) and fixation index (*F*_ST_) were calculated using the vcftools v.0.1.14, with a sliding window approach (100-kb windows with 50-kb step length) (Zhang et al., 2021). The parameters for the VCFtools program were as follows: “--fst-window -size 100,000 --fst-window-step 50,000”. The selected region was to apply the top significant 1% for *F*_ST_ and π ratio. The intersection selective regions considered as under strong selective sweep and subsequently examined for potential candidate genes.

### Functional enrichment analysis

Gene Ontology (GO) and KEGG pathways were retrieved to determine clusters of functionally related genes (Harris et al., 2004; Jiao et al., 2012). KEGG pathway enrichment analysis was performed using KOBAS 2.0 (http://kobas.cbi.pku.edu.cn/), and a corrected *P*-value 0.05 was set as the threshold. Then the genome database (http://animal.omics.pro/code/index.php/SheepVar) was used to retrieve the mutation frequency distribution of SNPs in the world's domestic sheep breeds, and analyzed the differences in mutation frequency.

### PCR for BRIP1 gene

Based on the results of whole genome resequencing in this study and combined with previous literature, a correlation was found between the *BRIP1* gene and breast cancer and ovarian cancer. Additionally, it was observed that the expression level of *BRIP1* in cervical cancer tissues is significantly lower than that in normal cervical tissues. Furthermore, upregulation of *BRIP1* gene expression has been reported to increase the risk of breast cancer and have a significant impact on the reproductive function of female mammals. Therefore, it is speculated that the *BRIP1* gene may be a potential candidate gene affecting litter size in sheep. In this study, we selected the *BRIP1* gene as a candidate gene affecting litter size, analyzed its polymorphism in the population of Dexin mutton and fine wool sheep, and investigated its association with litter size. *BRIP1* (Gene ID: 101107879) were designed with reference to the sheep. The PCR was performed in a 25 μL reaction volume containing 2 μL of DNA (50 ng), 1 μL of each primer, 12.5 μL of PCR Mix and 8.5 μL of water. The cycling protocol was 2 min at 95°C, followed by 36 cycles of 94°C for 30 s, TA C annealing for 30 s and 72°C for 45 s (TA for all markers are presented in Table 1).

**TABLE 1:** PCR primer sequences for *BRIP1*.

Loci	Sequence 5′-3′	Product length(bp)	Tm: °C
g:10972067 C > T	F:CTGATTTGGAGCCCTGAGTTA	546	61
	R:TCTCCTAGAATACACTTTGAACAT		
g:11098249 G > A	F:ACTGCTTGTTATGGGCTCTGAA	783	61
g:11098267 G > A			
g:11098431 G > A			
g:11098497 A > G			
g:11098533 A > G	R:TCAACTTCGTGTCTGTGCAGT		
g:11098537 T > C			
g:11098543 A > T			
g:11098683 G > A			

PCR-RFLP, analysis for TrKA, and NGF, genes.

The primers used in this study were designed with reference to the sheep *TrKA* (Gene ID: 101109763) and *NGF* (Gene Bank ID:101104540) mRNA sequences published in GenBank. Table 1 shows the primer sequence information. The PCR was performed in a 25 μL reaction volume containing 2 μL of DNA (50 ng), 1 μL of each primer, 12.5 μL of PCR Mix and 8.5 μL of water. The cycling protocol was 2 min at 95°C, followed by 36 cycles of 94°C for 30 s, TA°C annealing for 30 s and 72°C for 45 s (TA for all markers are presented in Table 2). Additionally, the gene polymorphism was detected by PCR-RFLP technique. The *TrKA* gene was digested by restriction enzyme Sac II, and the *NGF* gene was digested by restriction enzyme SfiI. In a 15 μL digestion reaction system, including 7 μL of PCR product, 1 μL of restriction enzyme *Sac II* or *Sfi I*, 1 μL of 10 × Buffer and 5 μL of ddH_2_O. Then water bath at 37°C for 5 h.

**TABLE 2:** PCR primer sequences for *TrKA* and *NGF*.

Gene	Sequence 5′-3′	Product length (bp)	Tm: °C
*TrKA*	F: CTCCCGACTCTCACCGCC	543	63
	R: CGAGGAAGCCAGCACAGA		
*NGF*	F: CCA​AGA​CTT​GCA​ATT​GTC​ACC	812	61
	R: TTA​CCT​TGA​CAC​AGT​CCT​CCG		

### Statistical analysis

Genotypic and allelic frequencies and the Hardy-Weinberg equilibriums were estimated using the SPSS 20.0 (IBM Corp., Armonk, NY, United States). The logistic regression model was used to analyse the association of polymorphisms with litter size in different sheep breeding farm: Y_ij_ = μ+F_i_ + G_j+_S_k_ + e_ijk_, in which Y was the phenotypic value of the litter size, μ was the overall population mean, F_i_ was the farm effect, G_i_ was genotype effect, S_k_ was the fixed effect of season (k = 1, 2, 3, 4), and e_ijk_ was the random residual. Additionally, the logistic regression model was used to analyse the association of polymorphisms with ovulation rate in different sheep populations: Y_ij_ = μ+G_i_ + e_ij_, in which Y was the phenotypic value of the ovulation rate, μ was the overall population mean, G_i_ was genotype effect, and e_ij_ was the random residual. Analysis was performed using the logistic regression model procedure of SAS (Ver 9.2; SAS Institute Inc., Cary, NC, United States). The data are expressed as the mean ± standard error (SE). Differences between means were accepted as statistically significant at *P* < 0.05 and as a trend at 0.05 < *P* < 0.10.

## Results

### Reads and mapping

**FIGURE 1:**
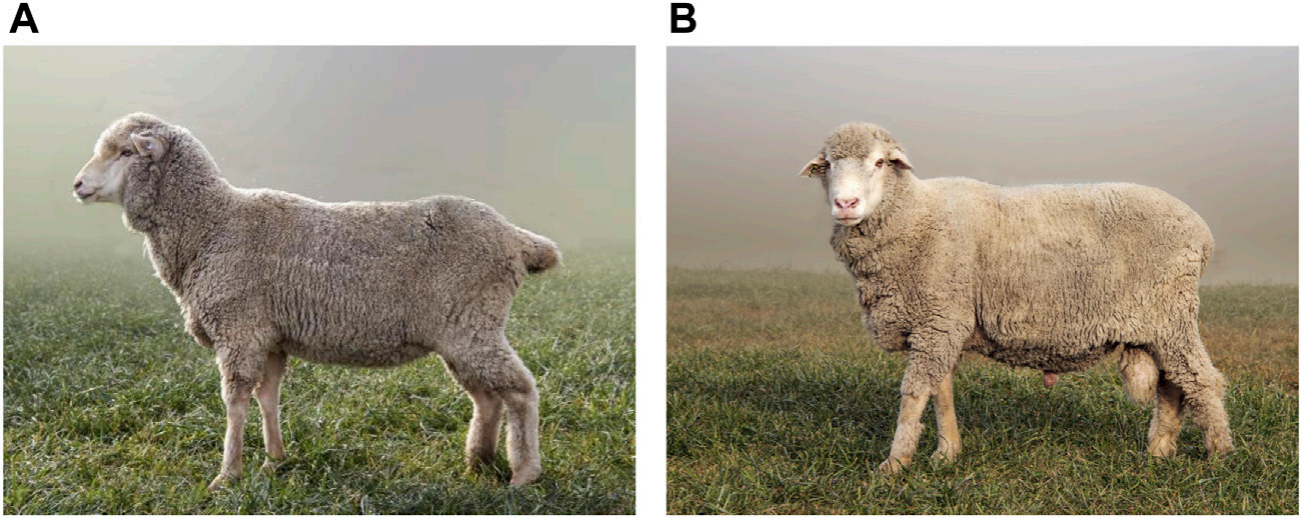
Female **(A)** and male **(B)** Dexin mutton and fine-wool sheep.

High-depth whole-genome resequencing of the 51 samples of Dexin mutton and fine-wool sheep (Figure 1; Supplementary Data S1) generated a total of 5,236.338G Gb, the ratio of the total sequenced bases to the genome size, that is, the minimum sequencing depth, was 4.32×; the maximum sequencing depth was 11.55×,the average sequencing depth was 7.45×, and an average genome coverage of 98.20% (Q20 ≥ 98.17% and Q30 ≥ 96.71%), The Mapping rate after alignment with the reference genome reached more than 99%. The GC base contents ranged from 42.10% to 45.34%, with an average of 43.24%. (Supplementary Data S1).

### Population genetic structure

**FIGURE 2:**
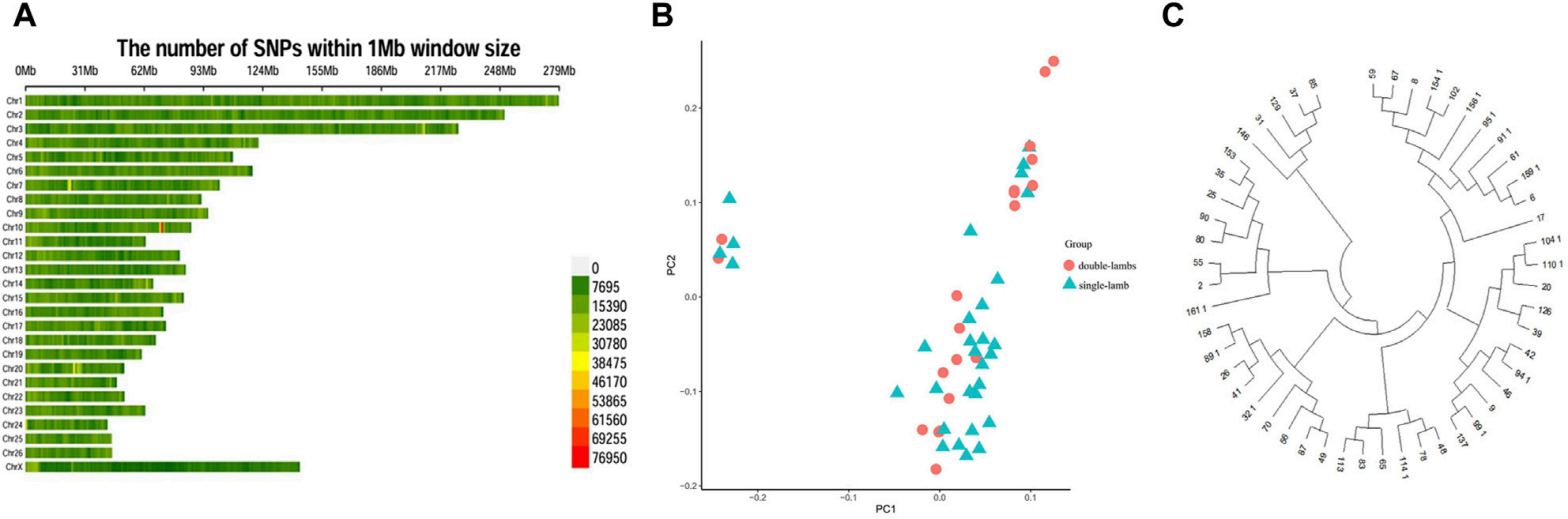
Population genetic structure. **(A)** Distribution of SNPs on chromosomes. **(B)** PCA analysis. **(C)** NJ tree.

SNPs had a different distribution on chromosomes (Figure 2A). We conducted a principal component analysis. PCA analysis provided corroborating evidence for the two groups. These two groups could not be distinguished, was gathered together (Figure 2B). For another, the NJ tree revealed that the two populations were clustered into one group, which was consistent with the PCA results (Figure 2C).

### Selective imprints of litter size trait of dexin mutton and fine-wool sheep

In order to screen candidate genes for litter size trait, the experimental population was divided into single-lamb group and double-lamb group according to the litter size record. Two methods of *F*_ST_ and Pi-ratio were used to screen candidate genes for reproductive traits and growth traits.

**FIGURE 3:**
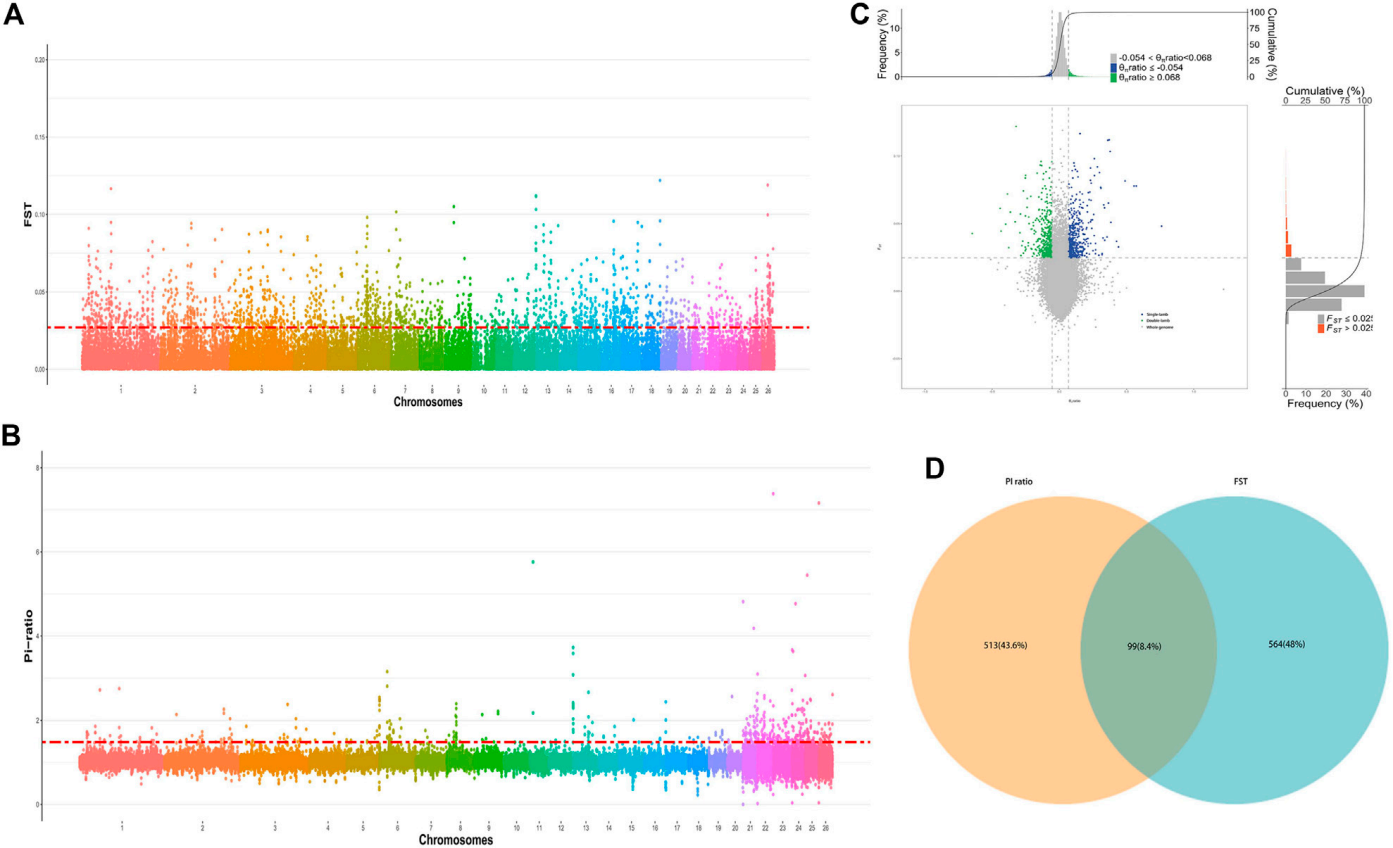
**(A)** The distribution of *F*_ST_ on autosomes of single lamb and double lamb of Dexin mutton and fine-wool sheep. **(B)** The distribution of Pi-ratio on autosomes of single lamb and litter size of Dexin mutton and fine-wool sheep. **(C)** Selection region and selection signal. **(D)** The intersection results of *F*_ST_ and Pi-ratio methods.

The top 1% region of *F*_ST_ (>0.027) and Pi-ratio (>1.48), and annotating the first 1% region by ANNOVAR (v.21-Jun-2013), 663 genes were retained in FST after removing duplicate genes (Figure 3A; Supplementary Data S2), and 612 genes were retained in Pi-ratio (Figure 3B; Supplementary Data S3). The intersection of *F*_ST_ and Pi-ratio annotated genes included 99 genes (Figures 3C, D).

Through the above two selection methods, we screened the corresponding signal regions in 26 autosomes of single and double lamb populations (Table 3). Consistent signal regions were found on chromosomes 11 and 12. For example, the position was in Chr 11: 10,900,000-10,950,000 region includes *BRIP1* and *INTS2* genes; the position was in Chr 12: 79,960,000–80,010,000 region includes *ELF3, COMMD8, LOC121816089, IP09, SHISA4, LOC121816077, LMOD1* genes. Additionally, this study also screened the “maker gene” *BMPR1B* which was significantly related to sheep reproduction.

**TABLE 3:** Candidate genes related to reproductive performance.

Chr	Gene
1	*ADAMTS1*
1	*NGF*
1	*TrKA*
3	*ACSS3*
6	*BMPR1B*
7	*BMP4*
11	*BRIP1*
17	*SDSL*

### Functional enrichment analysis

**FIGURE 4:**
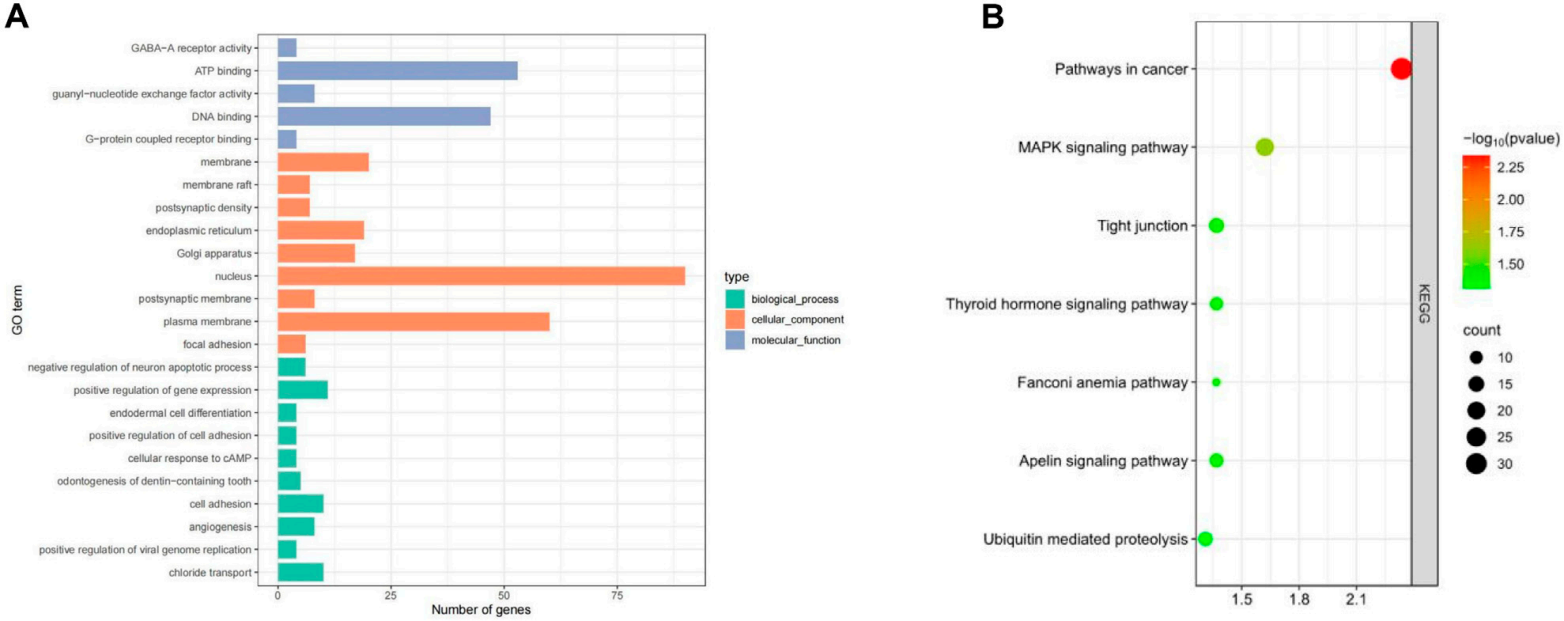
Enrichment results of candidate genes in Dexin mutton and fine-wool sheep. **(A)** GO Enrichment. **(B)** KEGG pathways.

GO enrichment and KEGG pathway analysis of the selected candidate genes were performed using DAVID and KOBAS. A total of 99 genes were annotated (*P* < 0.05) and enriched in important economic traits such as growth traits, reproductive traits, immune adaptability, etc. Notably, candidate genes were mainly enriched in ATP binding, DNA binding, G-protein coupled receptor binding, nucleus and positive regulation of gene expression, etc. Through the KEGG pathway analysis, candidate genes *BRIP1, NGF, ACSS3 BMP4, BMPR1B* and other genes were enriched in Fanconi anemia pathway, MAKP signaling pathway, Thyroid hormone signaling pathway and other signaling pathways. Some genes are also enriched in cancer-related signaling pathways such as Pathway in cancer (Figure 4).

### Candidate genes and mutation sites

**FIGURE 5:**
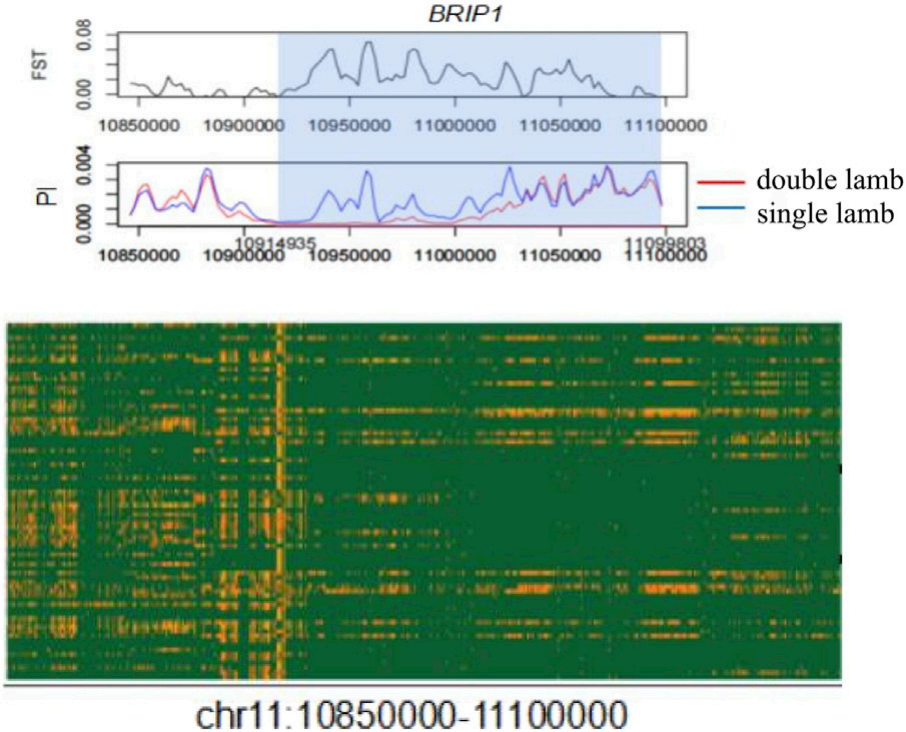
Local signal map and corresponding haplotype heat map of chromosome 11 region.

There were obvious signal differences and haplotype differences in the chromosome 11 region between single-lamb and double-lamb populations (Figure 5). The SnpEff software annotation showed that there were 9 missense mutation SNPs in the *BRIP1* gene, which were Chr 11:10,972,067 c.1136C > T p.Thr379Ile, Chr 11:11,098,249 c.2900G > A p.Arg967Gln, Chr 11:11,098,267 c.2918G > A p.Ser973Asn,Chr 11:11,098,431 c.3082G > A p.Asp1028Asn, Chr 11:11,098,497 c.3148A > G p.Asn1050Asp, Chr 11:11,098,533 c.3184A > G p.Ile1062Val, Chr 11:11,098,537 c.3188T > C p.Val1063Ala, Chr 11:11,098,543 c.3194A > T p.Glu1065Val, Chr 11:11,098,683 c.3334G > A p.Gly1112Arg.

**FIGURE 6:**
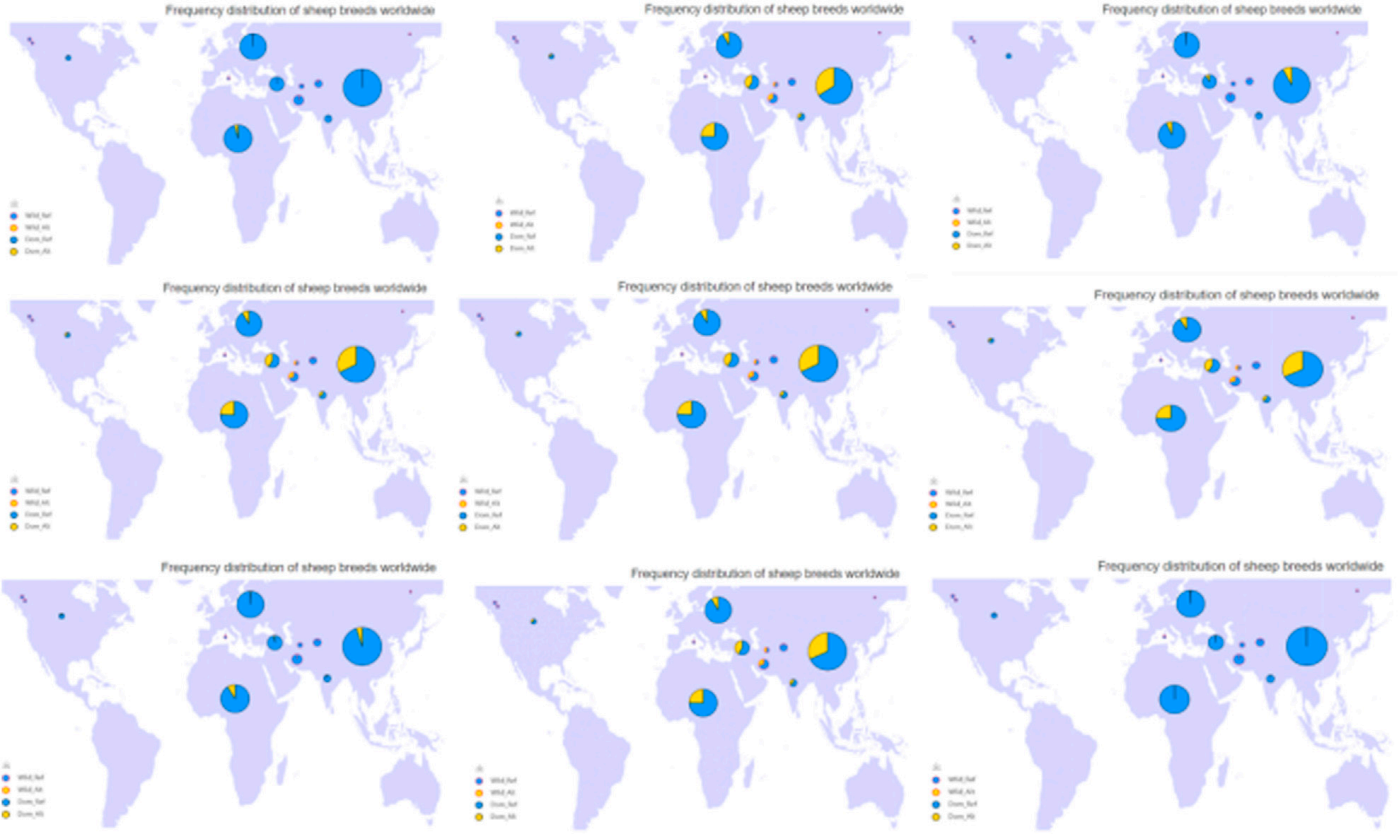
The frequency distribution map of in domestic sheep in the world.

The mutation frequency distribution of 9 missense mutations in the BRIP1 gene in the world domestic sheep breeds is shown in Figure 6 by sheep genome database (http://animal.omics.pro/code/index.php/SheepVar), and the mutation frequency in the world domestic sheep is quite different.

### Identification of litter size traits genes

**FIGURE 7:**
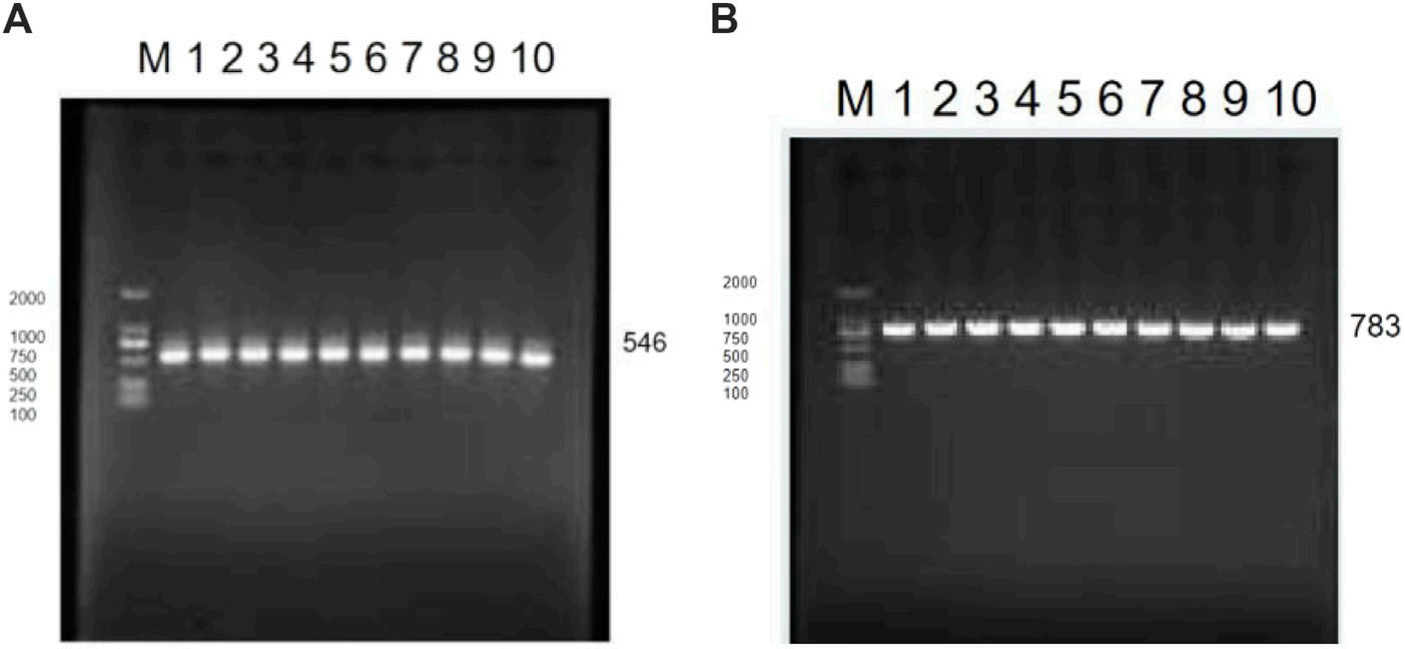
**(A,B)** PCR amplificationfor *BRIP1* gene.

**FIGURE 8:**
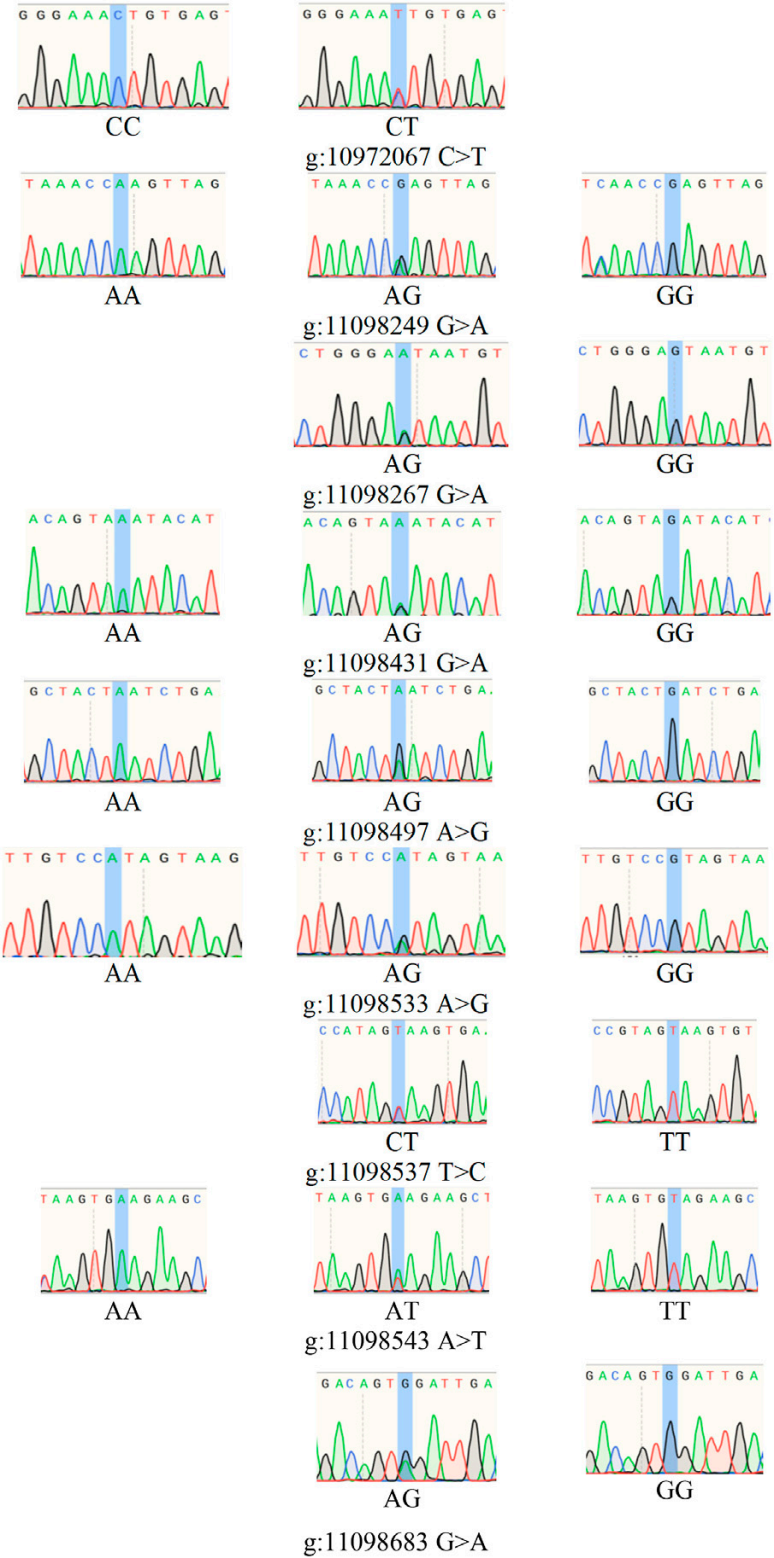
SNPs of *BRIP1* gene.

The PCR products of *BRIP1* (Figures 7A, B) gene was detected by PCR. The results showed that the size of the PCR products was consistent with the expected results. Then, the SNPs of *BRIP1* gene was detected by Sanger sequencing. 9 SNPs mutations were identified, and the results was shown in Table 5. Further, it was found that the g:11098497 A > G and g:11098533 A > G loci deviated from Hardy-weinberg equilibrium among the 9 SNPs mutation loci of *BRIP1* gene exon (*P* < 0.05) (Table 4). The sequencing results of different genotypes were shown in Figure 8.

**TABLE 4:** Population genetic analysis of the *BPIP1* gene.

Loci	Genotype	Genotype frequency	Allele frequency	He	Ho	Ne	PIC	χ2	*P*-Value
g:10972067 C > T	CC (108)	0.99	(C) 0.995	0.991	0.009	1.009	0.009	0.002	0.962
	CT (1)	0.01	(T) 0.005						
g:11098249 G > A	GA (21)	0.19	(G) 0.885	0.799	0.201	1.252	0.181	0.301	0.583
	AA (2)	0.02	(A) 0.115						
	GG (87)	0.79							
g:11098267 G > A	GA (3)	0.03	(G) 0.985	0.973	0.027	1.028	0.027	0.021	0.885
	GG (107)	0.97	(A) 0.015						
g:11098431 G > A	GA (21)	0.19	(G) 0.895	0.813	0.187	1.230	0.170	0.042	0.837
	GG (88)	0.80	(A) 0.105						
	AA (1)	0.01							
g:11098497 A > G	AA (88)	0.80	(A) 0.882	0.792	0.208	1.263	0.187	5.080	0.024
	GA (18)	0.16	(G) 0.118						
	GG (4)	0.04							
g:11098533 A > G	AA (88)	0.80	(A) 0.882	0.792	0.208	1.263	0.187	5.080	0.024
	GA (18)	0.16	(G) 0.118						
	GG (4)	0.04							
g:11098537 T > C	CT (5)	0.05	(C) 0.025	0.956	0.044	1.046	0.043	0.059	0.807
	TT (105)	0.95	(T) 0.975						
g:11098543 A > T	AA (88)	0.80	(A) 0.895	0.813	0.187	1.230	0.170	0.042	0.837
	AT (21)	0.19	(T) 0.105						
	TT (1)	0.01							
g:11098683 G > A	GA (3)	0.03	(G) 0.985	0.973	0.027	1.028	0.027	0.021	0.885
	GG (107)	0.97	(A) 0.015						

In this study, the association between the litter size and different genotypes of *BRIP1* gene g.11098497 A > G and g.11098533 A > G mutation sites of 255 Dexin mutton and fine-wool sheep was analyzed. The results are shown in Table 6. There was a strong linkage effect at the three mutation sites of *BRIP1* gene, and the litter size with GG and AG genotypes was significantly higher than that in AA genotypes (*P* < 0.05) (Table 5).

**TABLE 5:** Association analysis between genotypes of *BRIP1* of litter size.

Loci	Farm	Genotype	Number	Litter size
g:11098497 A > G	Wenquan	AA	108	1.77 ± 0.08^b^
		AG	31	2.16 ± 0.15^a^
		GG	14	2.50 ± 0.22^a^
	Tianxin	AA	49	1.36 ± 0.12^b^
		AG	42	1.98 ± 0.13^a^
		GG	4	1.75 ± 0.41^a^
g:11098533 A > G	Wenquan	AA	108	1.77 ± 0.08^b^
		AG	31	2.16 ± 0.15^a^
		GG	14	2.50 ± 0.22^a^
	Tianxin	AA	49	1.36 ± 0.12^b^
		AG	42	1.98 ± 0.13^a^
		GG	4	1.75 ± 0.41^a^

Note: Different lowercase letters represent significant differences (*P* < 0.05).

**FIGURE 9:**
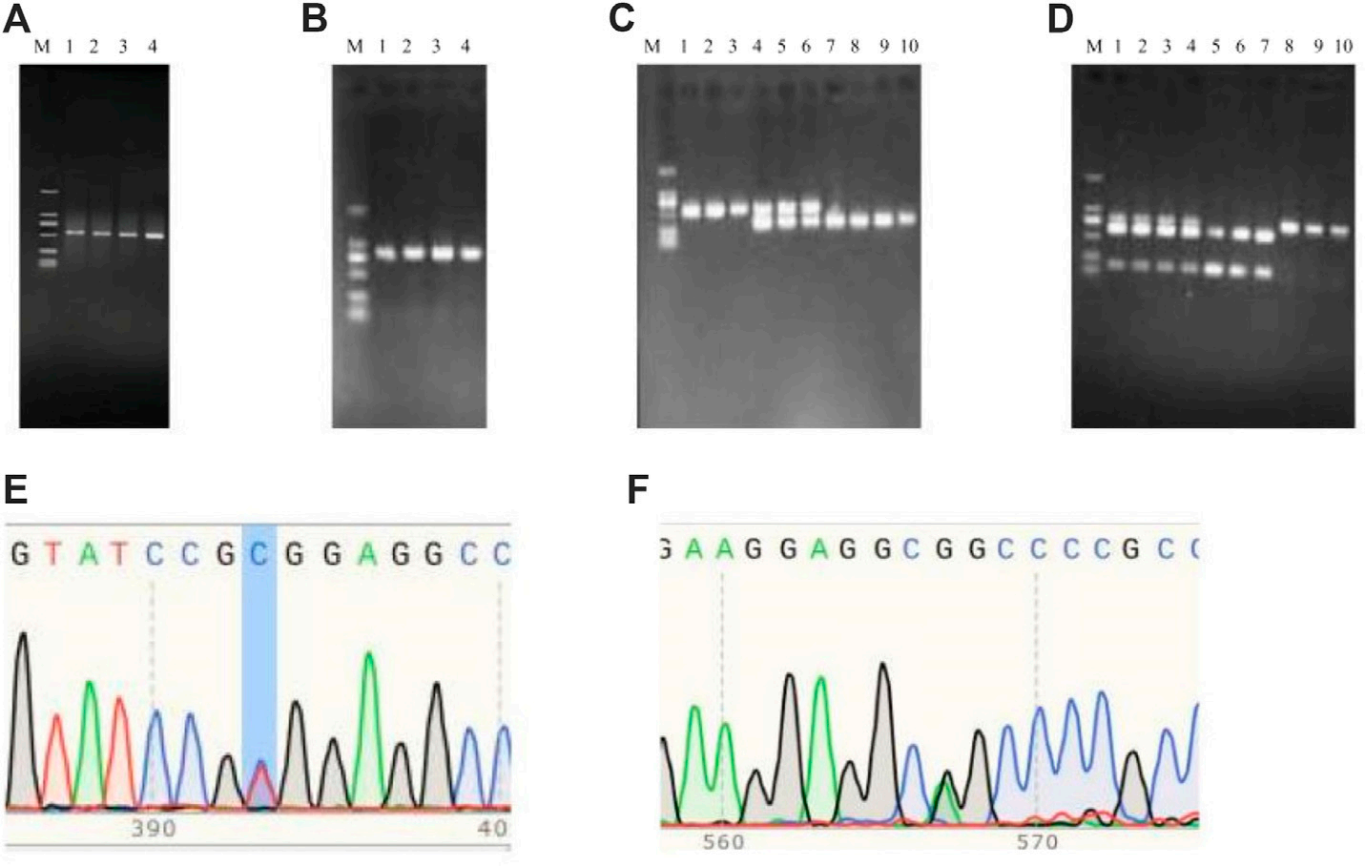
PCR amplification and PCR product sequencing peak diagrams and PCF-RFLP results of *TrKA* **(A,C,E)** and *NGF* **(B,D.F)**. M: MarkerD2K (The size of each band from bottom to top was 100, 250, 500, 750, 1,000, 2000, respectively).

The PCR products of *TrKA* (Figure 9A) and *NGF* (Figure 9B) genes were detected by PCR. The results showed that the size of the PCR products was consistent with the expected results. Using the PCR-RFLP method, the PCR product of *TrKA* digested by Sac II produced two bands of 432 bp and 111 bp, which were identified as CC genotype. A 543 bp band was identified as TT genotype. When the locus was in the CT heterozygous type, three bands of 543 bp, 432 bp, and 111 bp were produced, but the 111bp band was not shown because the 1% agarose gel had no obvious separation effect on the 111 bp band (Figure 9C). For NGF gene, the PCR product of *NGF* digested by Sfi I produced two bands of 205 bp and 607 bp, respectively, and the genotype was identified as GG type. A 812 bp band was identified as AA genotype. Three bands of 812 bp, 205 bp, and 607 bp were produced, and the genotype was identified as GA type (Figure 9D). Finally, the bands of the digested product were consistent with the expected size. The results of PCR sequencing peak map were also consistent (Figures 9E, F).

### Early breeding molecular markers for litter size traits

Hardy-Weinberg equilibrium test of *TrKA* gene showed that the locus was moderately polymorphic in both fields. The frequencies of CC, CT and TT genotypes in Tianxin breeding farm were 0.17, 0.33 and 0.51, respectively. The frequencies of CC, CT and TT genotypes in Wenquan breeding farm were 0.1, 0.45 and 0.45, respectively. TT was the dominant genotype. Chi-square test showed that the site was deviated from Hardy-Weinberg disequilibrium (*P*< 0.05) in Tianxin breeding farm, suggesting that the site had strong selection potential in Tianxin (Table 6). Hardy-Weinberg equilibrium test of NGF gene showed that the locus was in moderate polymorphism in both farms. The frequencies of GG, GA and AA genotypes in Tianxin breeding farm were 0.11, 0.19 and 0.71, respectively, and GG was the dominant genotype. The frequencies of GG, GA and AA genotypes in Wenquan breeding farm were 0.07, 0.24 and 0.68, respectively, and AA was the dominant genotype. Chi-square test showed that both Tianxin and Wenquan were deviated from Hardy-Weinberg disequilibrium (*P* < 0.01), suggesting that the locus had strong selection potential in this population (Table 7).

**TABLE 6:** Population genetic analysis of *TrKA* gene in different breeding farm.

Farm	Genotype frequency			Allele frequency		MAF	Ho	He	Ne	PIC	S	Chi-squared test
	CC	CT	TT	C	T							
Tianxin	0.17	0.33	0.51	0.33	0.67	0.33	0.56	0.44	1.8	0.35	0.64	6.61(*P* < 0.05)
Wenquan	0.10	0.45	0.45	0.33	0.67	0.33	0.56	0.44	1.78	0.34	0.63	0.17(*P* > 0.05)

**TABLE 7:** Population genetic analysis of *NGF* gene in different breeding farm.

Farm	Genotype frequency			Allele frequency		MAF	Ho	He	Ne	PIC	S	Chi-squared test
	GG	GA	AA	G	A							
Tianxin	0.11	0.19	0.71	0.2	0.8	0.2	0.68	0.32	1.47	0.27	0.5	15.81(*P* < 0.01)
Wenquan	0.07	0.24	0.68	0.19	0.81	0.19	0.69	0.31	1.46	0.26	0.49	7.48(*P* < 0.01)

Correlation analysis showed that the TT genotype of *TrKA* was the dominant genotype, which could increase the litter size. The GG genotype of *NGF* gene was the dominant genotype, which also increased the litter size (Table 8). The results showed that *TrKA* gene and *NGF* gene had an effect on the litter size of Dexin mutton and fine-wool sheep.

**TABLE 8:** Association analysis between genotypes of *TrKA* and *NGF* of litter size.

Gene	Farm	Genotype	Litter size
*TrKA*	Tianxin	CC	1.35 ± 0.50^b^
		CT	1.49 ± 0.50^ab^
		TT	1.79 ± 0.50^a^
	Wenquan	CC	1.46 ± 0.52^b^
		CT	1.78 ± 0.49^ab^
		TT	2.11 ± 0.41^a^
*NGF*	Tianxin	AA	1.29 ± 0.48^b^
		GA	1.45 ± 0.49^ab^
		GG	1.76 ± 0.50^a^
	Wenquan	AA	1.34 ± 0.50^b^
		GA	1.75 ± 0.47^ab^
		GG	2.05 ± 0.43^a^

Note: Different lowercase letters represent significant differences (*P* < 0.05).

## Discussion

Sheep was one of the earliest domesticated animals. It was not only providing important means of living such as meat, milk, skin and hair for human beings, but also was an important part of the spread of human civilization (Li et al., 2020). China had rich sheep variety resources and had good development potential. It occupied an important position in animal husbandry and played an important role in promoting the increase of farmers and herdsmen’s income. Since the late 1980s, China had vigorously developed animal husbandry and had become the country with the largest number of sheep breeding, slaughter and mutton production. However, on the whole, there was still a big gap between the mutton sheep industry and the developed countries. One of the main reasons was that the low fecundity of most sheep was one of the main factors limiting the development of sheep industry. While Litter size was an important economic trait affected by many factors such as heredity, and its heritability is low. It was impossible to achieve the effect of multi-gene polymerization by using traditional techniques to mine candidate genes affecting sheep litter size. Importantly, for some breeding programs of mutton sheep, litter size was one of the main selection objectives. Therefore, it was of great significance to reveal the genetic basis of reproductive traits by using whole genome resequencing technology to mine the selected regions and candidate genes of sheep litter size traits, and to clarify the germplasm characteristics.

Dexin mutton and fine-wool sheep is a new breed cultivated by our breeding team after 16 years in Xinjiang, China. German merino sheep was used as the female parent and Chinese merino sheep was used as the male parent. The breeding process was progressive hybridization, cross fixation and breeding improvement. The molecular breeding was combined to form a high prolificacy and fast growth and development mutton performance. In the study, we obtained a total of 5,236.338 Gb, with an average depth of 7.45× per individual and an average genome coverage of 98.20% (Q20 ≥ 98.17% and Q30 ≥ 96.71%), and the Mapping rate after alignment with the reference genome reached more than 99%. Additionally, the single lamb and double lambs were were clustered into one group. The PCA results were consistent with the NJ tree results. The important genes of litter size traits were identified by *F*_ST_ and Pi-ratio. A total of 99 genes were screened that may affect important economic traits such as growth, reproduction and immune adaptability of sheep. This was also consistent with the breeding process of Dexin mutton and fine-wool sheep.

The reproductive performance of sheep was an important factor affecting its breeding efficiency, and the litter size was an important trait to measure the reproductive traits of sheep. Improving the litter size of sheep was an important means to promote the vigorous development of sheep industry in China. Therefore, it is of great significance to identify the important genes of the litter size traits of the self-cultivated meat fine-wool sheep in Xinjiang. Interestingly, in the process of identifying the trait genes of litter size traits, we found that in addition to the maker gene “*BMPR1B*” which was significantly related to the litter size trait, *ACSS3, SDSL BMP4, ADAMTS1, NGF* and *BRIP1* were also found. These candiate genes mainly enriched in MAKP signaling pathway, Thyroid hormone signaling pathway and some genes were also enriched in cancer-related signaling pathways such as Pathway in cancer. MAPK signaling pathway and thyroid hormone signaling pathway play an important role in sheep reproduction (Su et al., 2023; Wang et al., 2023; Zhu et al., 2023). The MAPK signaling pathway was involved in regulating the physiological and biochemical processed of the sheep reproductive system, while the thyroid hormone signaling pathway affected the reproductive development and sex hormone regulation of sheep. The MAPK signaling pathway belonged to the classical conserved three-level enzymatic cascade reaction pathway, and its reaction mainly depended on the phosphorylation of protein kinases at different levels. MAPKKKs phosphorylate and activate specific MAPKKs, and MAPKKs then phosphorylate and activate downstream MAPKs, and then act on transcription factors to regulate specific gene expression (Wang et al., 2023). The previous study of the project team members found that miR-200c mediated MAPK8 to promote the development of sheep ovarian granulosa cells through MAPK signaling pathway and regulate the reproductive performance of sheep (Yang et al., 2016; Yang et al., 2018; Zhai et al., 2018; Zhu et al., 2022). Similarly, Hai (2023) demonstrated that leptin could inhibit the oxidation process of sheep endometrial epithelial cells through the MAPK pathway, thus playing a positive regulatory role in sheep embryo implantation. Lv et al. (2023) pointed that *UNC5C、BMPR1B* and *GLIS1* may affect the reproductive traits of sheep through MAPK signaling pathway. Yang and his team employed high-throughput sequencing approach to investigate genome-wide CNVs between Tibetan sheep and White Suffolk, found that thyroid hormone signaling pathway and *CTNNB1* gene that would be responsible for differential biological characteristics of breeds, especially reproductive traits (Yang et al., 2023).

Interestingly, *BRIP1* gene also called FANCJ. *BRIP1* gene-related reports are related to breast cancer and ovarian cancer. *BRIP1* was lowly expressed in cervical cancer tissues compared with normal cervical tissues and was closely related to poor prognosis. *BRIP1* gene was closely related to the function of breast cancer susceptibility *BRCA1* (breast cancer 1, early onset), also known as *BACH1* or *FANCJ*, located on 17q22-q24, about 18Kb, composed of 20 exons and 19 introns. *BRIP1* gene single nucleotide polymorphism could affect the susceptibility of cervical cancer, and *BRIP1* gene mutation was closely related to the occurrence and development of cervical cancer (Voutsadakis, 2020). Rafnar et al. (2011) confirmed that *BRIP1* gene was one of the representative tumor suppressor genes in ovarian cancer, suggesting that this mutation in BRIP1 gene may also increase the risk of other cancers. Similarity, it had also been reported that the expression of *BRIP1* was increased, which was easy to cause breast cancer and had a significant effect on the reproductive function of female mammals (Zou et al., 2016; Gupta et al., 2017). Moreover, this study also found that the frequency distribution of 9 SNPs of *BRIP1* gene was quite different in the world sheep. Additionally, g:11098497 A > G and g:11098533 A > G showed a strong linkage effect, the number of each genotype was the same, and the results of the association analysis between genotype and litter size were the same.

While, the *NGF* and *BMP4* genes were also found to be significantly correlated with reproduction traits. Nerve growth factor (*NGF*) played an important physiological role in the regulation of hypoxic-ischemic adaptation of central nervous system tissues or cells mainly by binding its high-affinity receptor *TrkA*. Abnormal expression of *NGF* and its receptors could cause diabetes, depression, Alzheimer’s disease and other diseases (Li et al., 2005; Wiener et al., 2015). In addition, the *NGF* gene also played an important role in some non-nervous systems such as reproduction and immunity. *NGF* was transported to the nucleus and binds to it by binding to cell surface receptors. So as to initiate cell activity, caused a series of biological effects, and played its function. Studies had shown that the synthesis of *NGF* in the ovary increases, which could increase the activity of the ovary and affect the reproductive performance of the female (Lansdown and Rees, 2012). *NGF* gene had a certain amount of expression in the animal reproductive system such as ovary, oviduct and uterus. It played an important physiological role in follicular growth and development, ovulation, ovarian hormone synthesis, gamete transport, sperm capacitation, fertilization and early embryos (Li et al., 2005; MTakeuchi and Harada, 2011). Also, a studies had found that *NGF* could promote the proliferation of granulosa cells and increase the pregnancy rate by inhibiting the downregulation of *CDKN1A* gene mediated by *ESR2* (Wang et al., 2015; Zheng et al., 2019). Naicy et al. found that the relative expression of *NGF* gene in the ovaries of prolific goats was significantly higher than that of singleton goats (Naicy et al., 2016). Herein, we showed that the TT genotype of *TrKA* was the dominant genotype, which could increased the litter size. The GG genotype of *NGF* gene was the dominant genotype, which also increased the litter size. The results showed that *TrKA* gene and *NGF* gene had an effect on the litter size of Dexin mutton and fine-wool sheep. Also, in the study, *NGF* (g: 92654175G > A) and *TrKA* (g: 106814725C > T) was transformed and applied, and it was found that the two SNPs in Wenquan Breeding Farm, Aksu, southern Xinjiang, China and Tianxin Breeding Farm, Aksu, southern Xinjiang, China could significantly increase litter size. The economic value of the current transformation was ¥50,000. Further transformation and application were in progress. Studies had also confirmed that the expression of *NGF* and the expression of *BMP4* had a synergistic effect, which promoted the ovulation of the female, thereby increased the number of lambs and improved the reproductive efficiency (Guang et al., 2010). Bone morphogenetic proteins (BMPs) are members of the transforming growth factor-β (TGF-β) superfamily and play an important role in the development process. *BMP4* was an important member of the BMPs family and a key gene in the TGF-β signaling pathway. It was widely expressed in organisms, and its biological functions involved almost all systems from embryo to adult, especially in reproductive activity (Hu et al., 2004). The function of the BMP family was mainly dependent on the classic BMP/Smad signaling pathway (Da Cunha et al., 2017). Xu et al. (2010) proved that *BMP4* could regulate ovulation rate via the BMP/Smad pathway and closely related molecules. Faure et al. found that the relative expression of *BMP4* in the sheep prolific group was significantly higher than that in the single group (Faure et al., 2005). With the deepening of research, the polymorphism of BMP4 gene had been proved to be associated with human diseases (Carstens and Michael, 2015), production performance of livestock and poultry (Fang et al., 2009). Fang et al. found that the polymorphism of *BMP4* gene was closely related to the growth and reproductive traits of goats (Fang et al., 2009). Similarly, Pan et al.found a missense mutation g.63454744T > G in the coding region of sheep *BMP4* gene by whole genome resequencing technology, which had a significant effect on the reproductive performance of ewes, which was consistent with the results of this study that *BMP4* gene may be an important gene affecting twinning traits (Zhangyuan et al., 2018). It was speculated that *BRIP1, NGF, BMP4* and other genes may regulate the growth, litter size traits of Dexin mutton and fine-wool.

As a new strain bred by German merino sheep was used as the female parent and Chinese merino sheep was used as the male parent, we used genome-wide resequencing to explore the selected regions for growth, reproduction, and immune adaptation, and identified the selection signals and important genes for litter size traits. The candidate genes were mainly enriched in MAPK signaling pathway, Fanconi anemia pathway, Thyroid hormone signaling pathway and other signaling pathways. It provides a new insights for molecular breeding of new breeds of Dexin mutton and fine-wool sheep, and also provides target genes and functional sites for the breeding of other new sheep breeds.

## Conclusion

We identified several major candidate genes and markers under selection in Dexin mutton and fine-wool sheep by whole genome re-sequencing. It played essential roles in reproduction, growth and immunity as well as other economic traits. We also explored a large number of genetic variants and genetic genetic basis underlying different phenotypes. Additionally, We identified *NGF, TrKA* and *BRIP1* genes was the important genes for sheep litter size traits. Our results contribute to understand the genetic basis of Dexin mutton and fine-wool sheep and provide valuable information for future breeding and improvement of new breeds. This study provided a comprehensive insight to the litter size traits of Dexin mutton and fine-wool sheep, which will underpin more-accurate identification of high prolific gene variants in the near future and facilitate novel breeding strategies.

## Data Availability

The data presented in the study are deposited in the NCBI, accession number PRJNA1084106.
